# Low Occurrence of Tuberculosis Drug Resistance among Pulmonary Tuberculosis Patients from an Urban Setting, with a Long-Running DOTS Program in Zambia

**DOI:** 10.1155/2010/938178

**Published:** 2010-06-30

**Authors:** Chanda Mulenga, Allan Chonde, Innocent C. Bwalya, Nathan Kapata, Mathilda Kakungu-Simpungwe, Sven Docx, Krista Fissette, Isdore Chola Shamputa, Françoise Portaels, Leen Rigouts

**Affiliations:** ^1^Biomedical Sciences Department, Tropical Diseases Research Centre, P.O. Box 71769, Ndola, Zambia; ^2^Mycobacteriology Unit, Institute of Tropical Medicine, Naionalestraat 155, B-2000 Antwerpen, Belgium; ^3^National Tuberculosis and Leprosy Program, Ministry of Health, P.O. Box 30205, Lusaka, Zambia; ^4^Ndola District Health Management Team, P.O. Box 70672, Ndola, Zambia; ^5^Tuberculosis Research Section, National Institutes of Health, LCID/NIAID, Bethesda, MD 20892, USA; ^6^Department of Biomedical Sciences, University of Antwerp, Campus Drie Eiken, Universiteitsplein 1, 2000 Antwerpen, Belgium

## Abstract

We set out to determine the levels of *Mycobacterium tuberculosis* resistance to first- and second-line TB drugs in an urban population in Zambia. Sputum samples were collected consecutively from all smear-positive, new and previously treated patients, from four diagnostic centres in Ndola between January and July 2006. Drug susceptibility testing was performed using the proportion method against four first- and two second-line TB drugs. *Results*. Among 156 new cases, any resistance was observed to be 7.7%, monoresistance to isoniazid and rifampicin was 4.5% and 1.3%, respectively. Of 31 retreatment cases, any resistance was observed to be 16.1%, monoresistance to isoniazid and rifampicin was 3.3% for each drug, and one case of resistance to both isoniazid and rifampicin (multidrug resistance) was detected. No resistance to kanamycin or ofloxacin was detected. *Conclusion*. Although not representative of the country, these results show low levels of drug resistance in a community with a long-standing DOTS experience. Resource constrained countries may reduce TB drug resistance by implementing community-based strategies that enhance treatment completion.

## 1. Introduction

Despite a long-running National Tuberculosis and Leprosy Program (NTLP), Zambia has seen a rapid increase in TB cases, especially after 1983, synchronous with the beginning of the HIV era in Zambia. The World Health Organization (WHO) estimates the prevalence of all forms of TB in Zambia at 707/100,000 and ranks Zambia as ninth in the world for TB incidence rate with an incidence of smear-positive cases at 280/100,000 [1]. 

Decentralization of the health sector in the late 1990s almost led to the collapse of the program, but its revival and recovery was realised through government's renewed focus and reorganization. Zambia adopted the Directly Observed Therapy Short Course (DOTS) strategy since 1993 and achieved 100% geographical DOTS coverage by 2004. According to the NTLP, in 2005, the Copperbelt and Lusaka provinces were responsible for 60% of the nation's notified cases and also showed some of the highest HIV prevalence rates at 17% and 20.8%, respectively [2]. Furthermore, a recent study in selected District Health clinics in Lusaka showed TB/HIV coinfection at 59% [3]. 

The prevalence of multidrug-resistant (MDR) TB in Zambia was determined to be relatively low in 2001; 1.8% and 2.3% in new and previously-treated cases, respectively [4]. Other reported data on TB drug resistance in Zambia stem from a survey in Zambian prisons in 2000–2001, which reported combined MDR among inmates at 9.5% [5]. Not-withstanding, because access to drug susceptibility testing (DST) is limited and not performed routinely, the picture of drug resistance in Zambia may be imprecise. This study was set out to document the prevailing drug resistance levels to four first-line drugs and two second-line drugs from an urban setting, where implementation of the DOTS strategy has been ongoing since the early 1990s.

## 2. Materials and Methods

### 2.1. Study Design

This is a part of a prospective cohort study in subjects with sputum smear-positive pulmonary TB (PTB).

### 2.2. Study Setting and Population

The study was conducted in health facilities in Ndola district under the Ndola District Health Management Team (DHMT). The Ndola DHMT has a catchment population of 374,757 persons [6] and 26 health centres, most of which are able to deliver TB treatment and care (treatment centres), but only six are able to perform smear microscopy (diagnostic centres). TB patients were treated according to the national TB guidelines [7], in line with the WHO treatment guidelines [8]. All new patients received an eight-month daily Category I regimen consisting of INH, RMP, pyrazinamide (PZA), and ethambutol (EMB) for two months followed by six months of INH and EMB. Patients who had previously taken TB drugs for more than one month received Category II treatment; INH, RMP, streptomycin (SM), EMB and PZA for two months daily followed by INH, RMP, EMB, and PZA for one month daily and INH, RMP, and EMB for five months administered daily. For this study, all consecutive previously-untreated and previously-treated smear-positive cases were enrolled from 4 of the 6 TB diagnostic centres. Data from the National Reference Laboratory for proficiency testing in smear microscopy show that, in 2006, 3 of the 4 participating diagnostic centres took part in the national program, with an average performance of 80% (75%, 80%, and 85%). The two clinics not included in the study were left out mainly because of their comparatively low population catchment areas at the time. All sputum smear-positive TB patients aged 15 years and above were included, whereas children below the age of 15 years were excluded. Taking into account WHO guidelines for resource-limited settings [9], sputum smear-negative TB patients were excluded as well.

### 2.3. Sample Size

Taking into consideration the 1.8% reporting national MDR prevalence among new TB cases and further assuming 15% losses that could arise from failed or contaminated cultures, a sample size of 344 would allow us to estimate the level of RMP resistance with a precision of 0.5% and 95% confidence interval.

### 2.4. Laboratory Methods

Sputum samples were stored in cetylpyridinium chloride (CPC) transport medium at ambient temperatures until the weekly collection to the Tropical Diseases Research Centre (TDRC). Samples were later transported to the reference laboratory in Lusaka and the Chests Diseases Laboratory (CDL) for culture on Löwenstein-Jensen (LJ) medium following decontamination using the Petroff method [10]. Cultures were incubated at 37°C and read weekly for growth for at least eight weeks. Successfully grown cultures were transported back to TDRC for storage and onward transportation of isolates to the Institute of Tropical Medicine (ITM, Antwerp, Belgium) for drug susceptibility testing (DST).

Isolates were identified as mycobacteria by smear microscopy and as *M. tuberculosis* by growth rate and temperature, colony morphology, and susceptibility to p-nitrobenzoic (PNB) acid [11]. Further identification was performed at TDRC using the Gen-Probe Accuprobe System for identification of *M. tuberculosis* complex (MTBC) and *M. avium-M. intracellular *complex (MAC) (Gen-Probe, San Diego, Calif.). 

Drug susceptibility testing was performed using the proportion method on LJ medium against four first-line drugs, that is, INH (0.2 and 1.0 *μ*g/ml), RMP (40 *μ*g/ml), SM (4 *μ*g/ml) and EMB (2 *μ*g/ml), and two second-line drugs,that is, ofloxacin (OFL; 2 *μ*g/ml) and kanamycin (KAN; 30 *μ*g/ml) [12]. The latter were chosen to detect possible extensively drug-resistant (XDR) TB, and pre-existing resistance to these drugs in the general population. Due to practical reasons, DST for PZA was not done. Detected RMP and INH resistance was further confirmed using the GenotypeMDRTB/*plus*/(Hain LifeScience, Nehren, Germany) following the manufacturers's instructions.

### 2.5. Genotyping of Failed Cultures for Drug Susceptibility

In addition, we were able to test a randomly selected subset of heat-inactivated bacterial suspensions that failed on subculture and subsequent DST at ITM and were kept at −20°C for DNA fingerprinting purposes. We performed sequencing of the rpoB gene according to Rigouts et al. [13] on 41 isolates. Further sequencing of the katG gene, according to Telenti et al. [14], was performed on isolates that revealed rpoB mutations.

### 2.6. Data Collection Methods

The study was conducted under routine TB care. Pulmonary TB patients were registered onto the TB program following sputum smear-positive microscopy using Ziehl Neelsen staining results. In the laboratory, all smear-positive samples were preserved in 1% CPC and periodically transported to the central laboratory for further processing. At the end of each day, data for all smear-positive samples was abstracted from the TB clinic register, into a register provided specifically for the study. Data routinely collected at diagnosis by the TB focal person included sociodemographic (name, sex, age, residence) and clinical (case type and microscopy result) data. Other data included treatment regimen, follow-up microscopy results and treatment outcome at the end of treatment. The study registers were checked against the clinic TB registers at the end of the study period to complete any missing data and for verification.

### 2.7. Ethical Consideration

Before commencement of the study, approval for the study protocol was obtained from the Ethics Committee at TDRC.

### 2.8. Statistical Methods

The data were double entered in Epi Info (Version 3.2.2, Centers for Disease Control and Prevention, Atlanta, GA, USA). All the electronic records were manually counterchecked against the source records for completeness and consistency. We performed data analysis using SAS (version 9.1.2, SAS Institute, Inc, Cary, NC, USA). The two-sided Pearson's asymptotic and exact chi square tests were appropriately used for comparisons to assess associations of sex, age, and treatment history using SAS 9.2 (SAS Institute Inc., Cary, NC, USA.) and StatXact 4.0.1 (Cytel Software Corp., Cambridge, MA, USA.). A *P*-value less than .05 was considered statistically significant.

## 3. Results

A total of 361 sputum smear-positive PTB subjects from the four selected diagnostic centres in Ndola from January to July 2006, were enrolled into the study. This represented 72% (361/499) of all the smear-positive PTB patients recorded in Ndola district during the same period. However, only 276 subjects yielded valid cultures and were identified as *M. tuberculosis* complex. Samples for the remaining 85 subjects, yielded either contaminated (*n* = 10) or negative (*n* = 75) cultures. A further 82 isolates had lost viability upon subculture of isolates for DST, and as a result, only 194 isolates were finally available for phenotypic DST. Additionally, we had to disqualify a result from the analysis because the patient was less than 15 years.

Of these 193 subjects, 132 were males and 61 were females, giving a male to female ratio of 2 : 1. The median age of these subjects was 31 (range: 15–79). Among these 193 subjects, 156 (80.8%) had never received TB treatment, while 31 (16.1%) were retreatment cases, and six (3.1%) had missing case type data. Comparison, for subjects recruited on the study, between the group for whom we obtained DST and those we could not obtain DST showed no statistical difference with regards to age (*P* = .999), sex (*P* = .467), and treatment history distribution (*P* = .999). 

As shown in Table 1, overall DST patterns showed that of the 193 subjects investigated, 17 (8.8%) were resistant to at least one of the four first-line drugs tested, and only one MDR case was detected. Resistance to INH was observed in 11 (5.7%), SM resistance in six (3.1%), RMP resistance in five (2.6%), and only one (0.5%) case showed resistance to EMB. Further, overall monoresistance was observed as follows: against INH in eight (4.1%), against RMP in three (1.6%), and against SM in two (1.0%) subjects.

### 3.1. Drug Resistance among New Cases

Among the 156 new cases, any resistance was observed in isolates from 12 (7.7%) subjects, of which monoresistance to INH was detected in seven (4.5%) subjects and monoresistance to RMP was detected in two (1.3%) subjects. One patient exhibited non-MDR polyresistance to SM and INH. There was no resistance observed against the two second-line drugs tested, OFL and KAN.

### 3.2. Drug Resistance among Previously Treated Cases

Among the 31 retreatment cases studied, 28 were relapse cases, one treatment failure and two defaulters. Of these, only five (16.1%) subjects showed any form of resistance to the four drugs, all being relapse cases. Mono-resistance was observed in one patient (3.3%) for INH and in another for RMP. Two subjects exhibited non-MDR polyresistance to SM and INH and to SM and RMP, while MDR was observed in another patient, who exhibited resistance to all four first-line drugs tested. Again, there was no resistance observed against OFL and KAN (Table 1).

### 3.3. Genotypic Drug Resistance

All isolates found to be RMP- and INH-resistant by phenotypic DST were confirmed to harbor mutations in the *rpo*B- and *kat*G genes, respectively. In addition, of the 41 cases that underwent sequencing of *rpo*B and *kat*G genes, two failed PCR or were too weak to yield valid sequencing results, whilst 39 yielded successful results. Of these 39 cases, 27 were new cases, 10 were previously treated cases and 2 had missing case-type data. Two (5.1%, 2/39) isolates were found RMP-resistant (1 Leu456 and 1 Glu438 mutation according to the *M. tuberculosis *nomenclature) of which one was found to be additionally INH resistant (Thr315 mutation). The former—likely RMP-monoresistant—subject was a 15-year-old new case, whereas the MDR subject was a 19-year-old relapse case.

## 4. Discussion

Our study revealed relatively low levels of any resistance to first-line drugs (8.8%) in Ndola, and for the first time systematically investigated and documented absence of resistance to second-line drugs (OFL and KAN). These levels of resistance to any of the first-line drugs are at the lower end of the spectrum when compared to the other 22 African countries reported in the WHO global project on anti-TB drug-resistance 2008 report, whose range is between 3.8%–39%. Further, compared to the only available countrywide drug-resistance data, for Zambia, the 2000 DR survey [4], MDR levels of 1.8% and 2.3% in new and previously treated cases, respectively, were reported, and we found MDR to be rare in this population. We cannot directly compare the results of the two; admittedly, the relatively low sample size of our study may have reduced the probability of picking up MDR cases and additionally, the two are different in coverage, one being nationwide and the other localized. But we cannot also exclude the fact that some of the isolates that did not grow in subcultures were MDR isolates, as it has been shown that some MDR isolates have reduced fitness [15]. However, among the 39 isolates that failed in phenotypic DST in our study, only one turned out to be MDRTB, suggesting that the proportion of MDR among the lost isolates was not significantly higher as compared to those with successful phenotypic DST (*P*-value = .241). It is possible that Ndola District, itself, may have low levels of resistance, being one of the earliest Districts to have implemented DOTS in Zambia. Through this long-standing experience, the Ndola District has benefited from the use of community volunteers and treatment supporters in their TB programme to assist in ensuring patients complete treatment, as evidenced by their relatively high treatment success rates of over 80% in 2008 (Provincial District Health Office). The WHO TB country profile data show treatment success rates for Zambia in 2006 to be 85% [16]. Data for Ndola District for that year were not available.

A successful national TB program will strive to avoid the emergence of drug resistance, particularly to the two most important anti-TB drugs, RMP and INH, to avoid development of MDR and eventually XDR *M. tuberculosis *strains. Unlike the earlier DR survey, which did not detect any RMP mono-resistance, this study, albeit with a relatively small sample size, detected RMP mono-resistance at 1.3% and 3.3% among new and previously-treated cases, respectively. Considering that over 96% of RMP-resistant cases can be detected by molecular tools [13], the rate might even be at 1.6% among new cases and drop to 2.4% among previously-treated cases if we include the subjects with only molecular analyses. Admittedly, we can not firmly conclude that this case was indeed RMP-mono-resistant as we did not test genes conferring resistance to SM and EMB, and as *kat*G mutations represent only between 50% and 95% of INH resistance [17]. Nevertheless, an unusually high rate of RMP mono-resistance of 8.9% was also observed in the prisons study mentioned earlier [5]_,_ and although this data was not confirmed by molecular techniques, these high levels of RMP-monoresistance might be attributed to high levels of HIV infection in prisons, reported in Zambia, at 26.7% [18]. These results may still imply an emerging undetected problem in the population or may indicate high transmission of RMP-mono-resistant strains among prisoners. 

Scrutiny of the available drug-resistance data in the African region, suggests that RMP-mono-resistance continues to be low. Of the 22 African countries reported in the WHO global project on anti-TB drug-resistance, only nine reported RMP mono-resistance in their population. Our results for RMP mono-resistance in new cases fall within the range of those reported by the WHO report for the 22 African countries (range 0.8% – 1.8%), but appears to be well above the figures reported by the 4 countries that reported RMP mono-resistance in retreatment cases (range: 0.8% – 1.3%) [4]. Our results were also higher than those reported in another noncountrywide survey in Bujumbura, 0.6% and 1.4% RMP mono-resistance in new and previously-treated subjects, respectively, with a combined resistance at 0.7% [19]. Another noncountrywide survey in Benin showed 2.2% of combined RMP mono-resistance [20]. Low RMP mono-resistance (1.0%) in retreatment cases was also reported in the Western Area and Kanema districts of Sierra Leone [21]. 

Until recently, RMP mono-resistance was rarely encountered worldwide. Knowledge of the mechanisms by which this resistance is developed remains vague. Multiple risk factors associated with mono-resistance to RMP have been suggested, including irregular drug intake, inadequate treatment of prior TB episode, prior history of TB, and prior use of rifamycins and rifabutin in treatment of TB and other bacterial infections [22–24]. Further, some studies have suggested that this type of resistance is rarely a result of transmission [25–27], while others show resistance in new cases as in our study, suggesting possible primary acquisition [19, 23]. Molecular typing results of this population are to be presented in another paper, but spoligotyping analysis did not indicate transmission of a single strain in samples exhibiting RMP mono-resistance. HIV disease has also been associated with the development of RMP mono-resistance due to malabsorption of anti-TB drugs [26, 28, 29]. Due to both nonavailability of routine VCT for TB patients at the time and the logistical limitations of the study, we were not able to determine the TB/HIV coinfection for the study population. Ndola's HIV prevalence in 2006 was estimated at 22.5% [30]. Additionally, DHMT data for Ndola district for 2008 cohort analysis indicate a 60.4% TB/HIV co-infection in smear-positive cases. RMP mono-resistance in immunocompromised populations may have detrimental consequences in terms of transmission and accumulation of polydrug resistance and MDR in a population [31]. 

On the other hand, INH mono-resistance seems to be more common globally, as was the case in our study. Our combined prevalence of 4.2% (4.5% and 3.3% in new and previously-treated cases) is within the range obtained in most of Africa. We observe a higher rate in new cases, contrary to what has been observed in most countries. Again, as mentioned earlier, HIV disease may play a role in this type of drug resistance [28]. 

Our study also detected two MDR cases of which one had full phenotypic DST results; this patient was resistant to all four first-line drugs. This case was a registered relapse case. However, after category II treatment, this case was declared cured for the second time, but died the following year. The cause of death could not be confirmed. 

No resistance against OFL and KAN was observed in this population, even in the MDR patient. This may be indicative of low use/access to these drugs in Zambia. So far, resistance to second-line drugs has been reported to be low in most African countries [32] except in some provinces of South Africa [4]. However, we must be wary that very few countries in Africa have tested these drugs [32], and the non-availability of data does not necessarily mean absence of resistance, and as such, mechanisms to monitor this trend should be encouraged. 

We acknowledge that the relatively high proportion of subjects for whom we could not obtain DST results due to negative culture or contamination could have introduced some bias. However, comparison of demographic characteristics of the two groups (those that had DST results and those without) did not show any statistical difference, and molecular analyses for part of the isolates with DST results showed only one additional MDR case and one mono-RMP case. Further, misclassification of patient history by clinic staff is possible, even though verification from subjects was obtained whenever possible. We were unable to obtain subjects' HIV test results for reasons mentioned earlier. Consequently, we are unable to link HIV status with drug resistance patterns observed in this population. Another limitation to our study is that due to logistical constraints smear-negative patients were excluded from the study. We acknowledge that in a high-HIV prevalence country like Zambia, smear-negative patients may contribute to the notifiable TB case load. However, there is no strong evidence to indicate that the proportion of cases that have DR varies substantially according to whether the TB case is smear-positive or smear-negative [9].

## 5. Conclusions and Recommendations

Although, the study may not be representative of the whole country, and the results are not necessarily comparable to previous data, our findings suggest that Ndola has maintained low levels of anti-TB drug resistance. These findings lend support to the notion that it is possible to keep TB drug resistance levels low even in resource constrained countries by implementing strategies that reduce treatment interruption. However, the appearance of mono-resistance to RMP, not previously reported in the general population, coupled with the sustained levels of INH resistance, may require further investigation.

## Figures and Tables

**Table 1:** Phenotypic drug resistance patterns to first-line and second-line antituberculosis drugs in 193 *M. tuberculosis* isolates from treated and previously treated subjects.

Resistance pattern	New cases *n* (%)	Previously treated cases *n* (%)	Missing information *n* (%)	Total *n* (%)
Total	156 (80.8)	31 (16.1)	6 (3.1)	193 (100)
Pan-susceptible	144 (92.3)	26 (83.9)	6 (100)	176 (91.2)
Any resistance	12 (7.7)	5 (16.1)	0	17 (8.8)
INH	8 (5.1)	3 (9.7)	0	11 (5.7)
RMP	2 (1.3)	3 (9.7)	0	5 (2.6)
SM	3 (1.9)	3 (9.7)	0	6 (3.1)
EMB	0	1 (3.2)	0	1 (0.5)
Mono-resistance				
INH	7 (4.5)	1 (3.2)	0	8 (4.1)
RMP	2 (1.3)	1 (3.2)	0	3 (1.6)
SM	2 (1.3)	0	0	2 (1.0)
EMB	0	0	0	0
Polyresistance				
MDR (INH+RMP+SM+EMB)	0	1 (3.2)	0	1 (0.5)
Non-MDR				
INH+SM	1 (0.6)	1 (3.2)	0	2 (1.0)
RMP+SM	0	1 (3.2)	0	1 (0.5)
				
OFLO	0	0	0	0
KAN	0	0	0	0

INH: isoniazid; RMP: rifampicin; SM: streptomycin; EMB: ethambutol; Oflo: ofloxacin; Kan: kanamycin, MDR, multidrug resistance.
